# Impact of non-surgical periodontal therapy on OHRQoL in an obese population, a randomised control trial

**DOI:** 10.1186/s12955-017-0793-7

**Published:** 2017-11-21

**Authors:** Samara S. Basher, R. Saub, R. D. Vaithilingam, S. H. Safii, Aqil M. Daher, F. H. Al-Bayaty, N. A. Baharuddin

**Affiliations:** 1 Department of Restorative Dentistry, Faculty of Dentistry, University of Malaya, Lembah Pantai, 50603 Kuala Lumpur, Malaysia; 2 Department of Community Oral Health & Clinical Prevention, Faculty of Dentistry, University of Malaya, Lembah Pantai, 50603 Kuala Lumpur, Malaysia; 3 Center of Periodontology Studies, Faculty of Dentistry, Universiti Teknologi MARA (UiTM), UiTM Campus Sg Buloh. Jalan Hospital, 47000 Sungai Buloh, Selangor Darul Ehsan Malaysia; 4 Community Medicine Unit, Faculty of Medicine and Defence Health, National Defence University of Malaysia, Sungai Besi Prime Camp, 57000 Kuala Lumpur, Malaysia

**Keywords:** Chronic Periodontitis, Obesity, Non-surgical periodontal therapy, OHRQoL, and OHIP-14

## Abstract

**Background:**

Oral Health Related Quality of Life (OHRQoL) is an important measure of disease and intervention outcomes. Chronic periodontitis (CP) is an inflammatory condition that is associated with obesity and adversely affects OHRQoL. Obese patients with CP incur a double burden of disease. In this article we aimed to explore the effect of Non-Surgical Periodontal Therapy (NSPT) on OHRQoL among obese participants with chronic periodontitis.

**Materials and Methods:**

This was a randomised control clinical trial at the Faculty of Dentistry, University of Malaya. A total of 66 obese patients with chronic periodontitis were randomly allocated into the treatment group (*n*=33) who received NSPT, while the control group (*n*=33) received no treatment. Four participants (2 from each group) were non-contactable 12 weeks post intervention. Therefore, their data were removed from the final analysis. The protocol involved questionnaires (characteristics and OHRQoL (Oral Health Impact Profile-14; OHIP-14)) and a clinical examination.

**Results:**

The OHIP prevalence of impact (PI), overall mean OHIP severity score (SS) and mean OHIP Extent of Impact (EI) at baseline and at the 12-week follow up were almost similar between the two groups and statistically not significant at (*p*=0.618), (*p*=0.573), and (*p*=0.915), respectively. However, in a within-group comparison, OHIP PI, OHIP SS, and OHIP EI showed a significant improvement for both treatment and control groups and the *p* values were ((0.002), (0.008) for PI), ((0.006) and (0.004) for SS) and ((0.006) and (0.002) for EI) in-treatment and control groups, respectively.

**Conclusion:**

NSPT did not significantly affect the OHRQoL among those obese with CP. Regardless, NSPT, functional limitation and psychological discomfort domains had significantly improved.

**Trial registration:**

(NCT02508415). Retrospectively registered on 2^nd^ of April 2015.

**Electronic supplementary material:**

The online version of this article (10.1186/s12955-017-0793-7) contains supplementary material, which is available to authorized users.

## Background

Chronic periodontitis (CP) is an inflammatory disease that adversely affects aesthetic, masticatory and speech functions of individuals [1, 2]. Oral health-related quality of life is an important measure of disease and intervention outcomes, which has become an important aspect that reflects patient satisfaction in relation to the specified domains of life [3–5]. Several studies documented that CP has a negative impact on OHRQoL [6–12]. Non-surgical periodontal therapy (NSPT) is considered to be the first-line of treating CP [13, 14]. NSPT was proven to be effective in treating periodontal disease [15–17]; Nonetheless, studies have shown a positive impact on OHRQoL following NSPT [18, 19]. Collectively, a systematic review by Shanbhag et al. investigated the impact of periodontal-disease treatment modalities on OHRQoL and concluded that NSPT can moderately improve OHRQoL in CP subjects, whereas other treatment modalities showed no significant difference [20].

Obesity was positively associated with periodontal disease and it was indicated as a risk factor for the development of CP [21]. Both experimental [22, 23] and observational [24–27] studies, which were confirmed with systematic reviews and meta-analysis [28, 29] ascertained the association of obesity and CP.

Oral health problems can affect a person’s perception of oral well-being, as well as their social and physical oral functioning. In addition, ORHQoL may indirectly affect obesity, difficulty in chewing may result in avoiding nutritious food like vegetables or might lead to overcooking, which reduces the nutrients and eventually leads to malnutrition or obesity [30, 31].

Owing to the fact that dental care constitutes a significant portion of yearly healthcare costs [32] accompanied by the increasing prevalence of obesity, obese individuals with CP incur a double burden of disease and a double jeopardy to their overall QOL.

The prevalence of being overweight and obesity is increasing with alarming figures in Malaysia, where it reached 33.6% and 19.5%, respectively [33]. Moreover, most studies of CP treatment with NSPT were done on the general population, and not exclusively in the obese. Thus, we felt the necessity of undertaking this study, particularly to patch the gap of knowledge regarding the impact of NSPT among obese individuals with CP.

Therefore, this study aims at assessing the impact of NSPT on OHRQoL among obese patients compared to controlled obese patients. We hypothesized that the treatment group will have improvement in ORHQoL.

## Methods

This was a randomized controlled clinical trial at the Faculty of Dentistry, University of Malaya. Ethical approval was granted by the Medical Ethics Committee, Faculty of Dentistry/University of Malaya (DFOP 1213/0079 (L)).The study was retrospectively registered in ClinicalTrials.gov as NCT02508415. Participants were recruited from October 1st, 2013 until April 30th, 2014. Obese is defined as an individual who has a BMI ≥ 27.5 kg/m2 [34].

### Participants

Patients with CP who attended the periodontal clinic at the Faculty of Dentistry were targeted in this study, those who fulfilled the inclusion and exclusion criteria were invited to participate in this study. The participants were screened for periodontal disease using a Basic Periodontal Examination (BPE).

The inclusion criteria included (i) Malaysians, (ii) BMI ≥ 27.5 kg/m^2^, (iii) aged≥ 30 years old, (iv) those who have at least 12 teeth and (v) diagnosed with CP. The exclusion criteria included those who (i) have received periodontal treatment within the past 6 months (ii), were on antibiotics within the past 4 months (iii), require prophylactic antibiotic coverage (iv), were on systemic or topical non-steroidal anti-inflammatory drugs (NSAIDs) for the past 4 months (v), are pregnant or intend to and lactating mothers (vi), are mentally handicapped, (vii) have rheumatic heart disease (viii), and had a valve replacement. Informed consent was obtained prior to commencement of treatment.

### Sample size

A total of 66 participants who were obese (BMI ≥ 27.5 kg/m^2^) and diagnosed with CP [35] participated in this study. The sample size was calculated based on the mean difference between test and control groups. We estimated that a total of 30 cases in each group was sufficient to detect a mean SS difference of 5 with 80% power of study. The parameters used in the sample size calculation were derived from the most comparable published data. Due to the anticipated 10% drop-out from previous studies [11, 36], the total sample size was 66.

### Randomization

Simple randomization technique was employed in this study using research randomizer software. Each participant was assigned a number from 1-66. A research assistant assigned the number to the participants to ensure complete concealment and optimum randomization. Then, the list was entered to the software that randomly allocate a specified number of participants into two groups; treatment and control.

### Data collection procedure

The procedure for clinical examination and NSPT was previously described by Akram et al. [9] in which demographic data and physical examination were assessed along with BPE. Participants were asked to complete OHIP-14 questionnaires with supervision of the research assistant. Periodontal parameters, including periodontal pocket depth (PPD) and recession (R) were carried out using a Williams Probe (Hu-Friedy, Chicago USA) at 6 sites per tooth. Clinical Attachment Loss (CAL) was calculated by the sum of PPD and R and was registered manually.

### Intervention

**Fig. 1:**
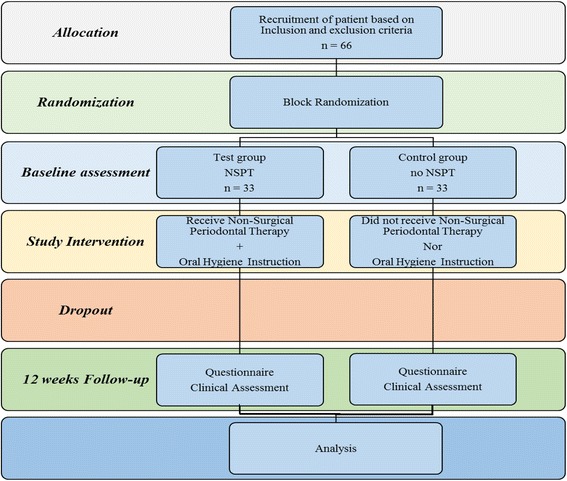
Flowchart showing the patients’ allocation and treatment intervention

The intervention group received an oral hygiene education, which included the use of a toothbrush, interdental brush and dental floss utilizing the modified Bass technique, and were also instructed to use a 0.12% Chlorhexidine mouth rinse. Scaling and root planning were conducted by the investigator within a single session using an ultrasonic scaler (SATELEC P5 Newtron XS, UK) and Gracey curettes (Hu-Friedy, Chicago, IL, USA). At 12 weeks, a recall visit was carried out in which re-motivation and professional prophylaxis was performed for the participants in the intervention group. On the other hand, the control group did not receive any oral hygiene education or NSPT and only an evaluation was performed including objective clinical and subjective OHRQoL assessment. Figure 1 outlines the study protocol.

### Outcomes

The OHRQoL was measured using the Oral Health Impact Profile-14 (OHIP-14), the shorter version of the Malaysian OHIP [37]. The questionnaire consisted of 14 questions based on 7 domains: functional limitations, physical pain, psychological discomfort, physical disability, psychological disability, social disability, and handicap. The participants were interviewed by trained interviewers for OHIP-14 to rate their OHRQoL based on a Likert scale ranging from (1) very often, (2) quite often, (3) sometimes, (4) seldom, (5) never, and (6) don’t know. Data input was carried out when (i) 20% of data (≥2 items from OHIP-14 questionnaires) were missing, (ii) blank entries or (iii) ‘don’t know’ responses, using all the mean scores of overall participants for each of the 14 questions. Three parameters of OHIP-14 were assessed, namely: prevalence of impact (PI), severity score (SS), and extent of impact (EI) [38]. PI is the percentage of participants reporting ≥1 impacts ‘very often/often’. SS is the sum of response code for 14 items, ranging from 14-70. Lower values indicate higher impact. EI is the number of items that were reported as very often/often for each subject. Both treatment and control groups completed the OHIP-14 question at baseline prior to administration of NSPT and during the 12 week follow up.

### Statistical analysis

All data were entered and analysed using SPSS version 20. Numerical variables were described with mean (± SD) and categorical variables with frequency and percentage. The association of paired categorical data was assessed with Cochrane’s Q test. A repeated measure ANOVA was used to test within- and between-group mean differences. The significance level was set at 0.05. Data was analysed with intention to treat strategy.

## Results

Socio-demographic characteristics of the participants are summarized in Table 1. Based on the characterisation of the participants, females were the predominant group with about 74% and 68% in the treatment and the control groups, respectively. The majority of the participants belonged to the Malay ethnic group, with 64.5% in the treatment and 77.4% in the control group. Equal distribution of participants was observed in primary, secondary, and tertiary education as well as others for both groups. The majority of the participants were non-smokers (84-91%) and non-alcoholic (84%) for both groups. The mean age was about 45 years for both groups. The mean BMI was about 32 to 35 kg/m^2^ for both groups. There was no significant difference between treatment and control groups, with regards to gender, ethnicity, levels of education, social habits, alcohol consumption, mean age, and BMI (*p* >0.05).

**Table 1:** Socio-demographic, habits and BMI data comparison between both groups

		NSPT	No NSPT	*p* value
		(*n*=31)	(*n*=31)	
		*n* (%)	*n* (%)	
Gender	Male	8 (25.80)	10 (32.25)	*0.576
	Female	23 (74.20)	21 (67.75)	
Ethnicity	Malay	20 (64.51)	24 (77.41)	*0.133
	Non-Malay	11 (35.49)	7 (22.59)	
Levels of education	Primary & Secondary	18 (58.06)	18 (58.06)	*1.000
	Tertiary & Others	13 (41.94)	13 (41.94)	
Smoking	Smoker	3 (9.67)	5 (16.12)	*0.635
	Non-smoker	28 (91.33)	26 (83.88)	
Alcohol	Yes	5 (16.12)	5 (16.12)	*1.000
	No	26 (83.88)	26 (83.88)	
Age mean (±)		45.03(10.72)	44.85 (9.02)	**0.052
BMI mean (±), kg/m^2^		32.98(4.93)	35.83(5.31)	**0.076

*Chi-square test

**Independent sample T-test

At baseline, periodontal parameters (PPD, R & CAL) showed no significant difference between groups. However, 3 months later, improvement in periodontal parameters was significant between treatment and control groups (*p* <0.05) (Table 2).

**Table 2:** Comparison of the periodontal parameters between treatment and control groups

	NSPT(*n*=31)	No NSPT (*n*=31)	*p value
Clinical Parameters			
Mean PPD (±)	4.19 (0.32)	4.36 (0.39)	0.079
Mean Recession (±)	0.57 (0.42)	0.65 (0.38)	0.456
Mean CAL (±)	2.89 (0.61)	3.06 (0.66)	0.285

*Independent sample T-test

Table 3 shows the comparisons for the within and between groups for OHIP-14 PI, SS and EI. At baseline, OHIP-14 PI, SS and EI were comparable for both groups. The overall OHIP PI was measured at baseline and 12 weeks for each group. The PI between groups were almost similar at baseline and 12 weeks post-NSPT (*p* =0.618). However, within-group comparison showed a significant difference for treatment (*p* =0.002) and control (*p*=0.008) groups. The overall mean OHIP SS increased significantly (*p* <0.05) in both treatment and control groups, however between groups difference was not significant (*p* =0.573). The overall mean OHIP EI was not statistically different at baseline and 12 weeks follow up for treatment and control groups, but it was reduced significantly within each study group 12 weeks later (treatment group *p* <0.006) and (control group *p* <0.002) (Table 3). Table 4 summarises changes in PI for all items at 12 weeks post treatment for both treatment and control groups. Most items showed non-significant reduction in PI. Nonetheless, a significant difference (*p* <0.05) was observed for bad breath and food impaction items. The attrition rate was 6%. Sensitivity analysis showed no difference in the results due to the attrition of 4 participants.

**Table 3:** Comparison of OHIP-14 PI, SS and EI within-group based on time and between groups

OHIP-14	Group	Time	outcome	*p* value	Difference	*p* value
OHIP PI n (%)	Treatment	Baseline	21 (67.74)	0.002*	11(35.48)	0.618^§^
		12 weeks	10 (32.26)			
	Control	Baseline	19 (61.29)	0.008*	7 (22.58)	
		12 weeks	12 (38.71)			
OHIP SS Mean (±SD)	Treatment	Baseline	57.20 (8.61)	0.006^#^	4.69(8.59)	0.573^§^
		12 weeks	61.89 (7.04)			
	Control	Baseline	58.29 (6.12)	0.004^#^	2.66(5.78)	
		12 weeks	60.95 (6.64)			
OHIP EI Mean (±SD)	Treatment	Baseline	1.62 (1.84)	0.006^#^	1.15(1.99)	0.915^§^
		12 weeks	0.47 (0.91)			
	Control	Baseline	1.50 (1.53)	0.002^#^	0.85(1.43)	
		12 weeks	0.65 (1.02)			

^*^ *p*value – Cochrane Q test

^#^within group *p*value – Repeated measure ANOVA

^§^between groups *p*value – Repeated measure ANOVA

**Table 4:** Comparison of OHIP-14 PI between baseline and 12 weeks post treatment

	Treatment			Control		
	Baseline n (%)	12 weeks n (%)	*p value	Baseline n (%)	12 weeks n (%)	*p value
Difficulty chewing	4 (12.90)	2 (6.45)	0.414	2 (6.45)	5 (16.12)	0.180
Bad breath	4 (12.90)	0 (0)	0.046^*^	5 (16.12)	0 (0)	0.025^*^
Discomfort eating	4 (12.90)	2 (6.45)	0.414	5 (16.12)	2 (6.45)	0.180
Ulcer	0 (0)	0 (0)	----	2 (6.45)	1 (3.22)	0.317
Food impaction	20(64.51)	8 (25.80)	0.002^*^	18 (58.06)	12 (38.70)	0.042^*^
Shy	6 (19.35)	2 (6.45)	0.157	3 (9.67)	0 (0)	0.083
Avoid certain food	2 (6.45)	2 (6.45)	1.000	5 (16.12)	1 (3.22)	0.102
Avoid smiling	2 (6.45)	0 (0)	0.157	1 (3.22)	2 (6.45)	0.564
Sleep disturbances	0 (0)	0 (0)	----	0 (0)	0 (0)	----
Concentration	0 (0)	0 (0)	----	1 (3.22)	0 (0)	0.317
Avoid going out	1 (3.22)	0 (0)	0.317	0 (0)	0 (0)	----
Daily activities	3 (9.67)	0 (0)	0.083	0 (0)	1 (3.22)	0.317
Spend money	3 (9.67)	2 (6.45)	0.564	3 (9.67)	1 (3.22)	0.157
Less confident	2 (6.45)	0 (0)	0.157	1 (3.22)	0 (0)	0.317

*Cochrane test

## Discussion

RCTs are considered to be the highest level of evidence that helps professionals evaluating intervention. The actual benefit of an intervention is measured to what extent it has impact on patient expectations and wellbeing.

The results of this study demonstrated that OHIP PI, SS and EI showed no substantial difference between treatment and control groups at baseline and 12 weeks later. However, among within-groups, there was a significant improvement of OHRQoL as measured with OHIP-14.

The actual change in patient perception was often attributed to the improvement of clinical status, which is objectively measured by the health profession. The conceptual model underlies the development of OHIP-14 and includes a biopsychosocial pathway in which the perception of QOL is related to health problems. Consequently, there are two different assessments involved: (i) measurement of clinical parameters by clinician, and (ii) measurement of OHRQoL as perceived by patients. The earlier measurement was based on signs and symptoms presented by patient, and clinicians use them as indicators of health and disease status. The latter measurement was based on what the patient perceives as affecting the OHRQoL. The patient-centred assessment is more salient to patients with CP where a patient’s concerns may differ from the traditional clinical endpoints [39, 40].

The results of this study came in line with other studies where the change in QOL occurred within-groups only. Although a longer study duration was expected to influence the outocme, two different educational programs were intorduced in a RCT, in which OHRQoL was improved after 12 months, but there was no difference between the two programs [41]. Similarly, a difference was not significant between the two groups, which were assigned different oral hygiene educational programs. Moreover, the intervention group showed better improvement [42]. Other studies showed similar results without a reasonable justification of such trends [43], [44].

CP is known for its silent disease nature and progresses slowly. Most patients might not be aware of suffering from CP because the condition does not commonly involve pain. The slow progressive nature of CP allows a patient to adapt to clinically presenting symptoms, such as food trapped and mobile tooth. Therefore, little or limited perception from patients to acknowledge CP as a condition may affect the OHRQoL.

A recent study on the same sample as reported by Akram et al. demonstrated that a within-group comparison showed improvement in all clinical periodontal parameters (Plaque score (PS), Gingival bleeding index (GBI), PPD and CAL) in both the treatment and control group at 12 weeks [45]. However, at 3 months, between-group comparison showed significant improvement in PS for the treatment group compared to the control group. NSPT was shown to be effective in improving clinical parameters among those obese with CP. Moreover, NSPT was shown to effectively improve PPD and CAL in shallow and moderately deep periodontal pockets, but not in deep periodontal pockets. Taken together, it is interesting to note that NSPT can provide better clinical outcomes, while the OHRQoL remains the same in that it does not have an impact on OHRQoL among those obese with CP.

It is interesting to note that both treatment and control groups presented with a high mean OHIP SS at baseline and 12 weeks post treatment. However, the differences in change for OHIP SS were significant (*p*<0.05). In the treatment group, the improvement observed could be attributed to the OHI and motivation as well as root surface debridement provided. Nevertheless, the improvement observed in the control group might be attributed to the tendency of persons to improve their performance when they are participating in an experiment. Individuals may change their behaviour due to the attention they are receiving from observers rather than because of any manipulation [46, 47].

The differences in change were based on participants’ perceptions and hence, these differences were probably much more relevant to the participants’ daily life. The mean EI at 12 weeks post NSPT showed significant reduction for both treatment and control groups at 0.47 (0.91) and 0.65 (1.02), respectively. These findings showed an overall improvement in the QoL (treatment: *p* < 0.006; control: *p* < 0.002), and this suggested that there was a significant improvement at the subject’s self-perception level of OHRQoL.

A detailed comparison of the OHIP-14 items revealed that only 2 out of 14 items were significantly reduced (*p*<0.05). The two items were ‘bad breath’ (functional limitation domain) and ‘food impaction’ (psychological discomfort domain). Previous studies in various populations were in agreement with the current findings, in terms of improvement in the ‘food impaction’ item [19] [48]. Improvement in ‘food impaction’ following NSPT in all the studies would be expected. Furcation involvement in molar teeth is a common clinical feature in moderate and severe CP. Similarly, as CP progresses, it involves alveolar bone resorption around the tooth, and wider interdental spaces will be observed that results in a food trap. Following NSPT, the severity of the condition may be reduced as those in the treatment group were now better able to clean the interdental areas using OHE knowledge. Other than the ‘psychological discomfort’ domain, the aforementioned studies also reported common impacts of OHIP PI related to physical pain following NSPT [19, 48]. In addition, Brauchle et al., also reported the positive impact of NSPT on OHRQoL at the social domain in those with CP [48].

Variation in response for specific item(s)/domain(s) involved between studies may be attributed to several factors. The OHIP questionnaire was originally developed using data from an oral health survey of older adults to examine the associations between OHIP scores and a variety of clinical indicators, such as tooth loss, caries and periodontal disease. In addition, OHIP-14 is not a condition-specific measure, i.e. the reported improvement in OHRQoL was not necessarily due to the resolution of the periodontal disease upon follow up [49]. Another possibility that the duration of 12 weeks was not enough to capture the changes were as seen in other studies where the follow up continued for approximately one year.

Previous studies raised concerns about difficulties in assessing the association between objective measures of dental diseases (e.g. dental caries and periodontal disease) and patient-based opinions of oral status. There was a weak relationship between the two and the objective measures may not accurately reflect patients' perceptions [49, 50]. These authors acknowledged that the results must be interpreted with care since the majority of the responses were unchanged with no impact on OHRQoL. In addition, prolonged exposure to CP could have led to a decrease in sensitivity of participants to the OHIP instruments. The level of awareness of patients regarding their CP status could also potentially influence the impact on responses. It could be anticipated that responses could differ in participants who are aware compared to those who are unaware of their periodontal condition [33]. It is possible that our Malaysian obese population could have (i) different aesthetic or social demands that dictate their perception towards disease, or (ii) do they pay attention to periodontal health since they were obese or possibly (iii) have more serious issues to deal with than their ‘less’ serious condition in the mouth.

It might be argued that our study is underpowered with a small sample size. Reports with similar outcomes recruited an almost comparable sample size with results consistent to this study [18, 19, 43, 51, 52]. Therefore, the argument might not be relevant.

Although we adopted a systematic approach in conducting the study and reporting the results, there were several inevitable limitations for this study: (i) failure of blinding the study groups because it involves active participation or otherwise. This might have caused a Hawthorn effect on the control group (ii), Impact of obesity on Quality of Life was not measured, (iii) In-line with other RCT, some ethical concerns were identified in regards to depriving the control group from the intervention, however, this was offset by the fact that those patients were not generally aware of their condition and the study offered them a diagnosis and treatment after the completion of the trial.

## Conclusion

NSPT does improve the OHRQoL in obese with CP; however, this clinical improvement was not statistically significant at a subjective level. Regardless, NSPT, functional limitation and psychological discomfort domains were significantly improved in obese with CP particularly in regards to the items, ‘food impaction’ and ‘bad breath’.

### Implications of the study

Few implications can be deduced from our study. Simple protective measures, such as NSPT can be provided at the primary dental care clinic and may be of supreme help to reduce the cost of treating CP as well as reducing the burden of disease. Assessment of QOL would be better combining specific and generic QOL measures. The results of this study add to evidence on the impact of NTSP on CP among obese patients and highlights that possible precautions need to be taken in similar studies. This study is reflective of a selected Asian population and could provide a path for future researchers to relate to our findings.

## Additional files

Additional file 1: Patient Information Sheet (ZIP 980 kb)

Additional file 2: Consent Form (ZIP 818 kb)

## Abbreviations

CAL: Clinical Attachment Level
CP: Chronic Periodontitis
EI: Extent of Impact
GBI: Gingival Bleeding Index
NSAIDs: Non-Steroidal Anti-Inflammatory Drugs
NSPT: Non-Surgical Periodontal Therapy
OHIP: Oral Health Impact Profile
OHRQoL: Oral Health-Related Quality of Life
PI: Prevalence of Impact
PPD: Periodontal Pocket Depth
PS: Plaque Score
SS: Severity Score
