# Supplementary material for: Impact of non-surgical periodontal therapy on OHRQoL in an obese population, a randomised control trial
Source: Health Qual Life Outcomes. 2017 Nov 21;15:225. doi: 10.1186/s12955-017-0793-7 (PMC5696769; doi:10.1186/s12955-017-0793-7)
Supplement: Supplementary file 1 — Patient Information Sheet (ZIP 980 kb) [file 12955_2017_793_MOESM1_ESM.zip › Patient Information Sheet Bahasa Melayu.pdf]

BORANG MAKLUMAT PESAKIT

Sila baca maklumat berikut dengan teliti, dan sekiranya ada apa-apa soalan, sila bincangkan dengan doktor berkenaan.

Tajuk Kajian:
Perkaitan antara Obesiti dan Penyakit Periodontik

Pengenalan:
Periodontitis (penyakit gusi) ialah penyakit yang mengakibatkan gusi berdarah dan kegoyahan gigi. Hanya sesetengah individu cenderung untuk mendapat penyakit gusi berbanding individu lain. Faktor keturunan (genetik) memainkan peranan penting dalam perkembangan penyakit ini. Kajian ini bertujuan mengenalpasti hubungkaitan faktor keturunan bagi penyakit gusi dengan faktor risiko, parameter mikrobiologi (kuman bagi penyakit gusi) dan kesannyakepada kualiti hidup pesakit. Pengenalpastian faktor keturunan yang terlibat dapat membantu kita memperbaiki atau mencipta cara-cara baru mengesan penyakit ini pada peringkat awal dan besar kemungkinan membolehkan kita memperkenalkan rawatan yang lebih berkesan. Oleh itu, anda dengan hormatnya dijemput mengambil bahagian dalam kajian kami.

Apakah tujuan kajian ini?
Tujuan kajian ini adalah untuk mengenalpasti terbitan gen-gen tertentu di dalam penyakit gusi dan hubungkaitnya dengan faktor-faktor risiko. Kami juga ingin menilai kesan penyakit periodontal ke atas kualiti hidup pesakit.

Apakah langkah-langkah perlu diikuti?
Kami memohon kebenaran anda untuk mengambil sampel darah, plak gigi dan cecair gusi. Kami juga memohon anda untuk mengisi borang kajiselidik yang mengandungi soalan berkaitan kualiti hidup. Semua maklumat yang dikumpul dan diproses akan disimpan sebagai maklumat sulit. Sebarang maklumat yang keluar daripada klinik akan dilindungi kerahsiaannya.

Siapakah tidak layak diterima untuk kajian?
Setiap individu yang mempunyai penyakit ini digalakkan menyertai dan tiada siapa yang akan dihalang daripada menyertai kajian ini.

Apakah manfaat kajian ini:
(a) kepada anda sebagai pesakit?
Penyelidikan ini akan membolehkan anda mengetahui status penyakit gusi anda. Anda akan diberi rawatan periodontium (gusi) di klinik periodontik yang melibatkan penskaleran dan penggilapan gigi (mencuci gigi) dan rawatan lanjutan bergantung kepada keseriusan penyakit gusi anda.

(b) kepada penyelidik?
Penemuan kajian ini dijangka akan menambah kefahaman tentang kaitan antara penyakit periodontal dan obesity di mana ia dapat membantu kami mengesan kejadian awal penyakit seterusnya memberikan rawatan yang bersesuaian kepada pesakit periodontal.

Apakah kemungkinan keburukan kajian ini?
Semasa pengambilan darah, anda mungkin akan mengalami sedikit ketidakselesaan fizikal.

Bolehkan saya menolak dari menyertai kajian ini?
Anda boleh tidak bersetuju untuk menyertai kajian ini kerana penglibatan dalam projek ini adalah secara sukarela. Anda tidak perlu memberi sebab, dan keputusan anda tidak akan mempengaruhi rawatan pergigian anda.

Siapakah patut saya berhubung sekiranya ada soalan tambahan sepanjang masa kajian ini?
Sila hubungi doktor anda. Sekiranya anda perlukan keterangan lanjut tentang kajian ini, sila hubungi kami pada bila-bila masa.

Nama Doktor:
Dr Nor Adinar bt Baharuddin Tel: 03-79674803 Telefon bimbit: 019-693-5088
