# Supplementary material for: Impact of non-surgical periodontal therapy on OHRQoL in an obese population, a randomised control trial
Source: Health Qual Life Outcomes. 2017 Nov 21;15:225. doi: 10.1186/s12955-017-0793-7 (PMC5696769; doi:10.1186/s12955-017-0793-7)
Supplement: Supplementary file 1 — Patient Information Sheet (ZIP 980 kb) [file 12955_2017_793_MOESM1_ESM.zip › Patient Information sheet English.pdf]

PATIENT INFORMATION SHEET

Please read the following information carefully. Do not hesitate to discuss any questions you may have with your doctor.

Study Title: Relationship between Obesity and Periodontal Disease

Introduction

Periodontitis (gum disease) is a disease which causes the gums to bleed and the teeth to become loose. It has been found that obese people are more likely to be affected by this problem than others. Fat cells secrete inflammatory mediators which in addition to the inflammatory mediators released by gum disease may lead to an increase in the amount of tissue breakdown in gum disease. This study attempts to investigate links in periodontal disease and its relationship to obesity as well as its impact on patient's quality of life. The identification of this relationship will help us to improvise or create new ways of detecting the disease at an earlier stage and perhaps enable a more effective treatment for this disease. Therefore, you are cordially invited to participate in our study into the nature of this disease.

What is the purpose of this study?

This study aims to identify the relationship between obese patients who have gum disease as well as its impact on the patient's quality of life.

What are the procedures to be followed?

We seek your permission to collect some samples of blood, dental plaque and fluid from the gums for the study.

We also require you to complete a set of questionnaire containing questions related to the quality of life. All the information collected and generated will be kept strictly confidential and any information that leaves the clinic will be anonymous so that you cannot be identified from it.

Who should not enter the study?

Every individual with the disease can volunteer to participate in the study. No one will be discriminated from participating in the study.

What will be the benefits of the study:

(a) To you as a subject?

This study will indicate the degree of gum disease present. You will receive gum treatment in the form of scaling and prophylaxis (cleaning of teeth) in the Periodontal Clinic, and if necessary other forms of gum treatment.

(b) To the investigator?

This study will provide us with a better understanding of the relationship between obesity and gum disease, which will help us in early detection and treatment of the disease in obese patients, thus enabling us to help other patients in future.

What are the possible drawbacks?

Some physical discomfort may result during drawing of blood.

Can I refuse to take part in the study?

Your participation is totally voluntary. You need not explain why you prefer not to take part in the study and it will not affect your dental treatment.

Who shall I contact if I have additional questions during the course of the study?

The best person to consult will be your Doctor who will be working with us closely. Should you need to find out more about the details of the study, you are welcome to contact us at any time.

Doctor's Name:
Dr Nor Adinar bt Baharuddin Tel: 03-79674803 Handphone:019-693-5088
