# Supplementary material for: Impact of non-surgical periodontal therapy on OHRQoL in an obese population, a randomised control trial
Source: Health Qual Life Outcomes. 2017 Nov 21;15:225. doi: 10.1186/s12955-017-0793-7 (PMC5696769; doi:10.1186/s12955-017-0793-7)
Supplement: Supplementary file 2 — Consent Form (ZIP 818 kb) [file 12955_2017_793_MOESM2_ESM.zip › Consent Form Bahasa Melayu.pdf]

KEIZINAN OLEH PESAKIT UNTUK PENYELIDIKAN KLINIKAL, *FAKULTI PERGIGIAN, UM, K.L.*

Saya, ... No. Kad Pengenalan... ....

(Nama pesakit)

beralamat..

(Alamat)

dengan ini bersetuju menyertai dalam penyelidikan klinikal (pengajian klinikal/pengajian soalselidik/percubaan ubat-ubatan) disebut berikut:

Tajuk Penyelidikan : **Perkaitan antara Obesiti dan Penyakit periodontik**

yang mana sifat dan tujuannya telah diterangkan kepada saya oleh Dr...

(Nama & jawatan doktor)

mengikut terjemahan...yang telah menterjemahkan kepada saya dengan

(Nama & jawatan penterjemah)

sepenuh kemampuan dan kebolehannya di dalam bahasa/loghat... .

Saya telah diberitahu bahawa dasar penyelidikan klinikal dalam keadaan metodologi, risiko dan komplikasi (mengikut kertas maklumat pesakit). Selepas mengetahui dan memahami semua kemungkinan kebaikan dan keburukan penyelidikan klinikal ini, saya merelakan/mengizinkan sendiri menyertai penyelidikan klinikal tersebut di atas.

Saya faham bahawa saya boleh menarik diri daripada penyelidikan klinikal ini pada bila-bila masa tanpa memberi sebarang alasan dalam situasi ini dan tidak akan dikecualikan dari doktor yang merawat.

Tarikh .... Tandatangan/Cap jari...

(Pesakit)

DI HADAPAN

Nama ....,

No. K/P ...., Tandatangan ....

Tarikh...

(Saksi untuk tandatangan pesakit)

Jawatan ....

Saya sahkan bahawa saya telah menerangkan kepada pesakit tentang sifat dan tujuan penyelidikan klinikal tersebut di atas.

Tarikh .... Tandatangan ....

(Doktor yang merawat)

KEIZINAN OLEH PESAKIT UNTUK PENYELIDIKAN KLINIKAL

No. Pend.
Nama
Jantina
Umur
Unit
