# Supplementary material for: Impact of non-surgical periodontal therapy on OHRQoL in an obese population, a randomised control trial
Source: Health Qual Life Outcomes. 2017 Nov 21;15:225. doi: 10.1186/s12955-017-0793-7 (PMC5696769; doi:10.1186/s12955-017-0793-7)
Supplement: Supplementary file 2 — Consent Form (ZIP 818 kb) [file 12955_2017_793_MOESM2_ESM.zip › Consent Form English.pdf]

CONSENT BY PATIENT FOR CLINICAL RESEARCH FACULTY OF DENTISTRY, UM, K.L.

I, ……………………………………………………………………………….. Identity Card No ……………………………………………………………….. .
(Name of patient)
of……
(Address)

hereby agree to take part in the clinical research (clinical study) specified below :

Title of Study :
Oral Health-Related Quality Of Life of Obese With Periodontal Disease Following Non-Surgical Periodontal Therapy

The purpose of this study has been explained to me by Dr ……………………………………………………………………………………….
(Name & designation of doctor)
and interpreted by ………..
(Name & designation of interpreter)
to the best of his/her ability in……………………. language/dialect.

I have been told about the methodology, possible adverse effects and complications regarding this clinical research through the patient information sheet. After knowing and understanding all the possible advantages and disadvantages of this research, I allow / permit myself the clinical research mentioned above.

After knowing and understanding all the possible advantages and disadvantages of this clinical research, I voluntarily consent of my own free will to participate in the clinical research specified above.

I understand that I can withdraw from this clinical research at any time without assigning my reason whatsoever and in such a situation shall not be denied the benefits of usual treatment by the attending doctors.

Date ……………………..………… Signature or thumbprint…………………………………
(Patient)

IN THE PRESENCE OF

Name ………………………………………………,
I/C No. ………………………………………………..,
Designation ……………………………………..

Signature ……….. ……………………………………….
(Witness for signature of patient)
Date: ……………………………………..

I confirm that I have explained to the patient the nature and purpose of the above mentioned clinical research.

Date …………………………..... Signature ……………………………………………………..
(Attending doctor)

**CONSENT BY PATIENT FOR CLINICAL RESEARCH** R.N:	R.N:<br>Name:<br>Sex:<br>Age:<br>Unit:
